# Characterization of UDP-Glucuronosyltransferases and the Potential Contribution to Nicotine Tolerance in *Myzus persicae*

**DOI:** 10.3390/ijms20153637

**Published:** 2019-07-25

**Authors:** Yiou Pan, Pengjun Xu, Xiaochun Zeng, Xuemei Liu, Qingli Shang

**Affiliations:** 1 College of Plant Science, Jilin University, Changchun 130062, China; 2 School of Agricultural Science, Zhengzhou University, Zhengzhou 450001, China; 3 Institute of Tobacco Research, Chinese Academy of Agricultural Sciences, Qingdao 266101, China

**Keywords:** UDP-glucuronosyltransferase, host adaptation, *Myzus persicae*, nicotine tolerance

## Abstract

Uridine diphosphate (UDP)-glycosyltransferases (UGTs) are major phase II detoxification enzymes involved in glycosylation of lipophilic endobiotics and xenobiotics, including phytoalexins. Nicotine, one of the most abundant secondary plant metabolites in tobacco, is highly toxic to herbivorous insects. Plant-herbivore competition is the major impetus for the evolution of large superfamilies of *UGTs* and other detoxification enzymes. However, UGT functions in green peach aphid (*Myzus persicae*) adaptation are unknown. In this study, we show that UGT inhibitors (sulfinpyrazone and 5-nitrouracil) significantly increased nicotine toxicity in *M. persicae nicotianae*, suggesting that UGTs may be involved in nicotine tolerance. In total, 101 *UGT* transcripts identified in the *M. persicae* genome/transcriptome were renamed according to the UGT Nomenclature Committee guidelines and grouped into 11 families, UGT329, UGT330, UGT339, UGT341–UGT345, and UGT348–UGT350, with UGT344 containing the most (57). Ten *UGTs* (*UGT330A3*, *UGT339A2*, *UGT341A6*, *UGT342B3*, *UGT343C3*, *UGT344D5*, *UGT344D8*, *UGT348A3*, *UGT349A3*, and *UGT350A3*) were highly expressed in *M. persicae nicotianae* compared to *M. persicae* sensu stricto. Knockdown of four UGTs (*UGT330A3*, *UGT344D5*, *UGT348A3*, and *UGT349A3*) significantly increased *M. persicae nicotianae* sensitivity to nicotine, suggesting that *UGT* expression in this subspecies may be associated with nicotine tolerance and thus host adaptation. This study reveals possible *UGTs* relevant to nicotine adaptation in tobacco-consuming *M. persicae nicotianae*, and the findings will facilitate further validation of the roles of these *UGTs* in nicotine tolerance.

## 1. Introduction

The peach potato or green peach aphid *Myzus persicae* Sulzer (Hemiptera: Aphididae) is a globally important pest that affects a broad range of agricultural and horticultural crops, causing significant damage both through direct feeding and transmission of many plant viruses [1]. The ability of *M. persicae* to adapt to new host plants has led to the formation of host races. The best documented case of this phenomenon is the adaptation of *M. persicae* to the host plant tobacco (*Nicotiana tabacum* L.); this led to the host race designated *M. persicae nicotianae* [2]. *M. persicae nicotianae* is morphologically and genetically distinct from *M. persicae* sensu stricto (s.s.). However, there are clear examples of gene flow between the two taxa [3]. In another case, *M. persicae* clones collected from Western Australia have adapted to feed successfully on *Lupinus angustifolius* (narrow-leafed lupine), and this adaptation might be due to enhanced tolerance to lupanine in the diet compared to that of non-adapted clones [4].

Plants produce many toxic secondary metabolites, some of which are thought to act in direct defense against herbivores by reducing their performance, survival, and reproduction. Furthermore, phytophagous insects have evolved various strategies to cope with allelochemicals, including detoxification enzymes, such as cytochrome P450 monooxygenases (P450), carboxylesterases (CarE), glutathione S-transferases (GSTs), and uridine diphosphate (UDP)-glycosyltransferases (UGTs) [5,6,7]. Various reports have shown that P450s of the CYP3 clade are involved in oxidative detoxification of furanocoumarins, alkaloids, and numerous other plant secondary metabolites and synthetic insecticides [8,9,10,11,12]. Nonetheless, the roles of *UGTs* in xenobiotic tolerance have not been sufficiently studied.

UGTs catalyze conjugation of a diverse range of small lipophilic xenobiotics and endobiotics with sugars to produce glycosides, which are water-soluble and can be efficiently excreted [13]. Therefore, glycosylation of toxins by UGTs is a particularly important detoxification mechanism [14,15]. However, only a few reports to date have indicated the involvement of insect UGTs in the detoxification of plant secondary xenobiotics, such as in *Manduca sexta* [16], *Helicoverpa assulta* [17,18], *Spodoptera littoralis* [19], and *Helicoverpa armigera* [20]. It has recently been demonstrated that increased expression and amplification of the *CYP6CY3* gene in *M. persicae nicotianae* plays a major role in detoxifying nicotine and that 2-hydroxynicotine and 5-hydroxynicotine are produced by hydroxylation reactions [21,22]. UGTs usually catalyze the transfer of glycosyl groups of sugar donors (e.g., UDP-glucose) to hydroxyl/amino groups of lipophilic compounds to produce glycosides [19,20], yet it remains unknown whether abundant *UGTs* are involved in nicotine tolerance via secondary detoxification metabolism in *M. persicae nicotianae*.

To advance our understanding of the potential roles of *UGTs* in nicotine tolerance in *M. persicae nicotianae*, the following experiments were performed: (1) *UGT* transcripts were identified in the *M. persicae* genome and transcriptome; (2) using quantitative real-time polymerase chain reaction (qRT-PCR), the expression profiles of *UGTs* that were identified in the transcriptome were analyzed in three *M. persicae* clones; and (3) the involvement of *UGTs* in nicotine tolerance in *M. persicae nicotianae* was functionally confirmed by RNA interference (RNAi) assays. In the present study, we potentially identified putative *UGT* transcripts in this subspecies and assigned standard names according to the guidelines of the UGT Nomenclature Committee. Our data provide preliminary insight into changes in *UGTs*’ expression in three *M. persicae* clones based on transcriptome data and their involvement in nicotine tolerance. These results may facilitate further study of the functions of *UGTs* in *M. persicae* with regards to host plant adaptation.

## 2. Results

### 2.1. Synergism Bioassays

**Figure 1:**
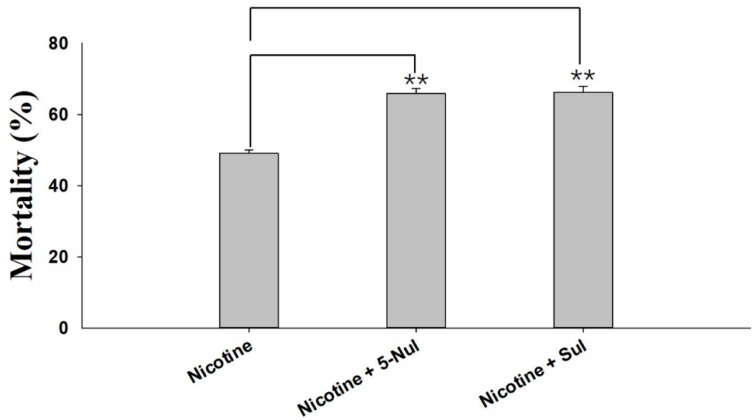
Synergistic effects of sulfinpyrazone (Sul) and 5-nitrouracil (5-Nul) on the toxicity of nicotine in *M. persicae nicotianae*. The final concentrations of nicotine, Sul, and 5-Nul in the artificial diet were 100 mg/L, 12.5 mg/L, and 12.5 mg/L, respectively. Mortality was recorded after 48 h of treatment (three replicates, 80 apterous adult aphids per replicate). Error bars indicate 95% confidence intervals (*n* = 3). ** Significant difference according to Student’s *t*-test (*p* < 0.01).

Two UGT inhibitors, sulfinpyrazone (Sul) and 5-nitrouracil (5-Nul), were used to analyze the possible roles of UGTs in nicotine tolerance. The results of the synergistic effects of 5-Nul and Sul on nicotine toxicity in *M. persicae nicotianae* are presented in Figure 1. 5-Nul and Sul possibly increased nicotine toxicity in apterous adult *M. persicae nicotianae*; mortality increased from 49.07% under nicotine treatment alone (100 mg/L) to 65.86%/66.13% under nicotine treatment (100 mg/L) with 5-Nul/Sul (12.5 mg/L) treatment (Figure 1). These results indicate that UGTs may be involved in nicotine tolerance in *M. persicae nicotianae*.

### 2.2. Identification and Phylogenetic Analysis of M. persicae UGTs

**Figure 2:**
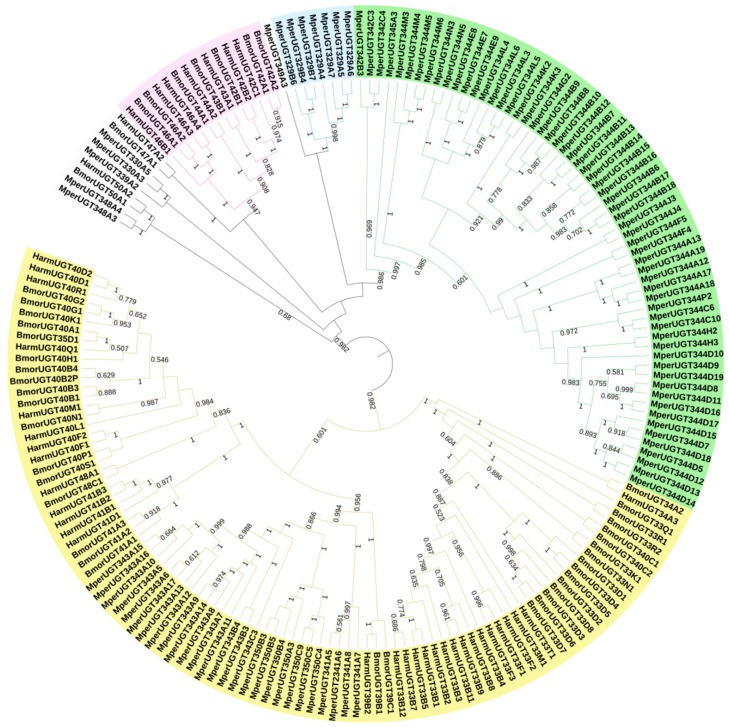
Phylogenetic analysis of *M. persicae* UGT genes and orthologs in *H. armigera* and *B*. *mori*. A phylogram was generated using the maximum likelihood method in MEGA7, and bootstrap values were computed based on 1000 replicates with a cutoff of <50%. The sequences used for constructing the tree are listed in Supplementary Data 1 and Supplementary Data 2.

Based on the transcriptome data for *M. persicae* (the clean reads obtained in this study were submitted to the NCBI/Sequence Read Archive (SRA) database under the SRA experiment Accession Number SRX1499035), 39 *UGT* transcripts with full-length or nearly full-length open reading frames (ORF) were identified, and these *UGTs* were named according to the UGT Nomenclature Committee (http://prime.vetmed.wsu.edu/resources/udp-glucuronsyltransferase-homepage) guidelines to include the following components: the symbol UGT, a family number, a subfamily letter, and an individual gene number. UGT families are defined as having 40% amino acid sequence identity, and subfamilies are defined as having 60% or greater amino acid identity [23]. The GenBank accession numbers are listed in Supplementary Data 1. Ninety *UGT* transcripts in the *M. persicae* genome were downloaded from the National Center for Biotechnology Information (NCBI, https://www.ncbi.nlm.nih.gov/nucleotide/) and named according to the UGT Nomenclature Committee guidelines; these *UGT* sequences were identified as representing 71 *UGT* transcripts. In total, 101 *UGT* transcripts were identified in *M. persicae* after removing redundant sequences (Supplementary Data 1). Phylogenetic analysis was used to evaluate the evolutionary relationships of *UGTs*. *UGT* sequences from *H*. *armigera* and *Bombyx mori* in the UGT Nomenclature Committee database (Supplementary Data 2) and *UGT* gene sequences from *M. persicae* were used to construct a phylogenetic tree (Figure 2). ClustalW was employed to align the amino acid sequences in MEGA7 software (http://www.megasoftware.net/), and the neighbor-joining method with 1000 bootstrap replicates was applied for construction of the phylogenetic tree. Bootstrap values above 50% are indicated on the branches.

The 101 *M. persicae UGT* transcripts were distributed into 11 families. Of the total, 57 *UGTs* were grouped into the UGT344 family; 16 into the UGT343 family; 7 each into the UGT329 and UGT350 families; 4 into the UGT341 family; 3 into the UGT342 family; 2 each into the UGT330 and UGT348 families; and only one each into the UGT339, UGT345, and UGT349 families (Figure 2, Supplementary Data 1). The gene evolution relationships of *H. armigera*/*B. mori* and *M. persicae*’s UGTs are far from each other. Because the roles of UGTs in xenobiotic compound tolerance are still poorly understood, we performed further analyses.

### 2.3. Conserved Domains of the M. persicae UGT Proteins

**Figure 3:**
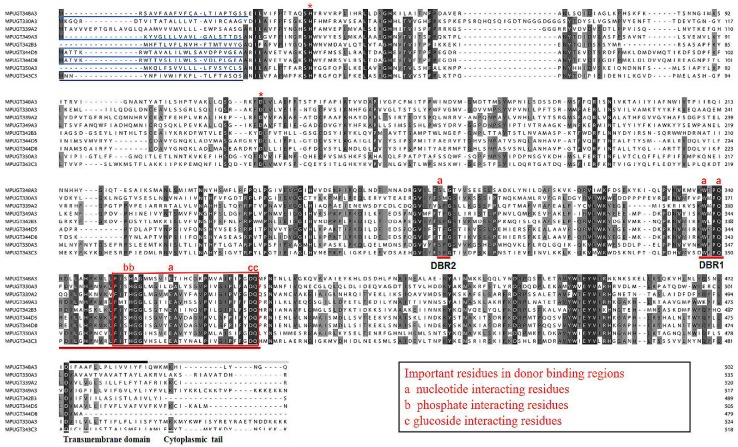
Alignment of the amino acid sequences of nine UGT genes from *M. persicae*. The predicted signal peptides in the N-terminus are underlined. The UGT signature motif is boxed. The transmembrane domains in the C-terminal half and the cytoplasmic tails are indicated with black and gray lines above the alignment. The important catalytic residues, H and D, are indicated with asterisks (*) above the alignment. The red lines under the alignment indicate the two donor-binding regions (DBRs), and several residues interacting with the sugar donor are indicated by letters (a, b, and c) above the alignment.

Multiple alignments of eight representative *M. persicae* UGT amino acid sequences revealed two major domains: a highly-variable N-terminal substrate-binding domain and a conserved C-terminal sugar donor-binding domain (Figure 3) [18]. All the UGTs contained a variable-length amino acid signal peptide found at the N-terminus, which was presumably cleaved after integration into the endoplasmic reticulum (ER) compartment. The two predicted sugar donor-binding regions (DBR1 and DBR2) and important residues interacting with the sugar donor and catalytic residues were conserved. The UGT motif signature sequence, (FVA)-(LIVMF)-(TS)-(HQ)-(SGAC)-G-X (2)-(STG)-X(2)-(DE)-X(6)-P-(LIVMFA)-(LIVMFA)-X(2)-P-(LMVFIQ)-X(2)-(DE)-Q, is present in the middle of the C-terminal domain, which shows high conservation. The alignment data suggest that these *M. persicae* UGT proteins may have glycoside conjugation activity.

### 2.4. Differences in UGT Gene Expressions among Three M. persicae Subspecies

**Figure 4:**
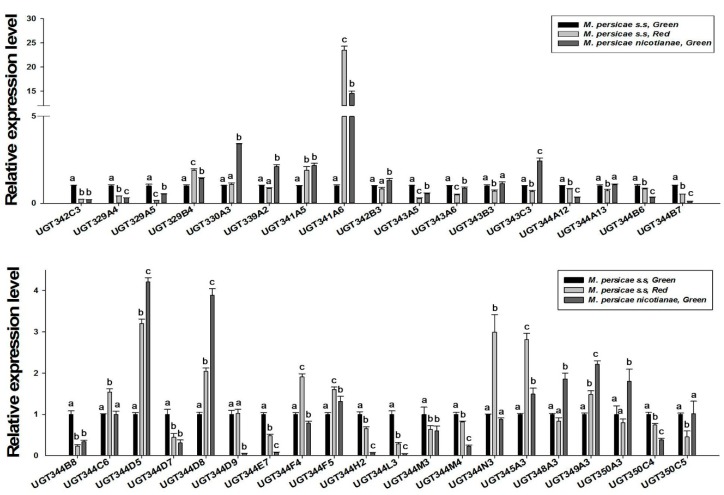
Relative expression levels of *UGTs* in three populations of *M. persicae* determined by real-time PCR. Actin and *para* (a voltage-gated sodium channel) were used as internal reference genes. Error bars indicate 95% confidence intervals (*n* = 3). Different letters on the bars indicate significant differences based on ANOVA followed by Tukey’s HSD multiple comparison test (*p* < 0.05).

qRT-PCRs were employed to determine the mRNA expression levels of 37 *UGTs* identified in the transcriptomes of three *M. persicae* subspecies. Expression of nine *UGTs* (*UGT330A3*, *UGT339A2*, *UGT342B3*, *UGT343C3*, *UGT344D5*, *UGT344D8*, *UGT348A3*, *UGT349A3*, and *UGT350A3*) was significantly higher in *M. persicae nicotianae* (green) than in *M. persicae* sensu stricto (green) and *M. persicae* sensu stricto (red) (Figure 4). Additionally, expression of *UGT341A6* was 0.62- and 14.55-fold higher in *M. persicae nicotianae* (green) than in *M. persicae* sensu stricto (green) and *M. persicae* sensu stricto (red), respectively. With regard to the other *UGTs*, levels were less significantly different or were not significantly different among the aphids (Figure 4).

### 2.5. Knockdown of UGT Transcripts Increases Mortality in M. persicae nicotianae

**Figure 5:**
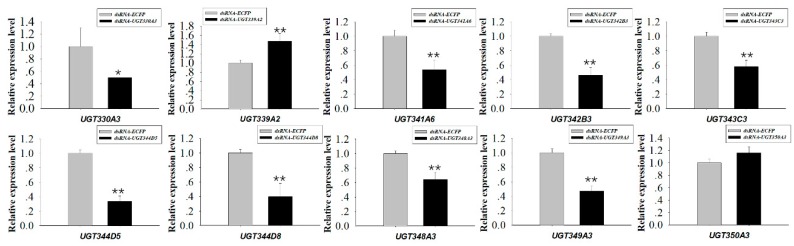
Relative expression of 10 *UGT* transcripts in *M. persicae nicotianae*. dsRNA-mediated 48 h after oral delivery of dsRNA. Suppression of *UGT* (*UGT330A3*, *UGT339A2*, *UGT341A6*, *UGT342B3*, *UGT343C3*, *UGT344D5*, *UGT344D8*, *UGT348A3*, *UGT349A3*, and *UGT350A3*) expression in *M. persicae nicotianae* fed an artificial diet with corresponding dsRNA (100 ng/μL) for 48 h. Error bars indicate 95% confidence intervals (*n* = 3). * Significant (*p* < 0.05) difference according to Student’s *t*-test. ** Significant (*p* < 0.01) difference according to Student’s *t*-test.

**Figure 6:**
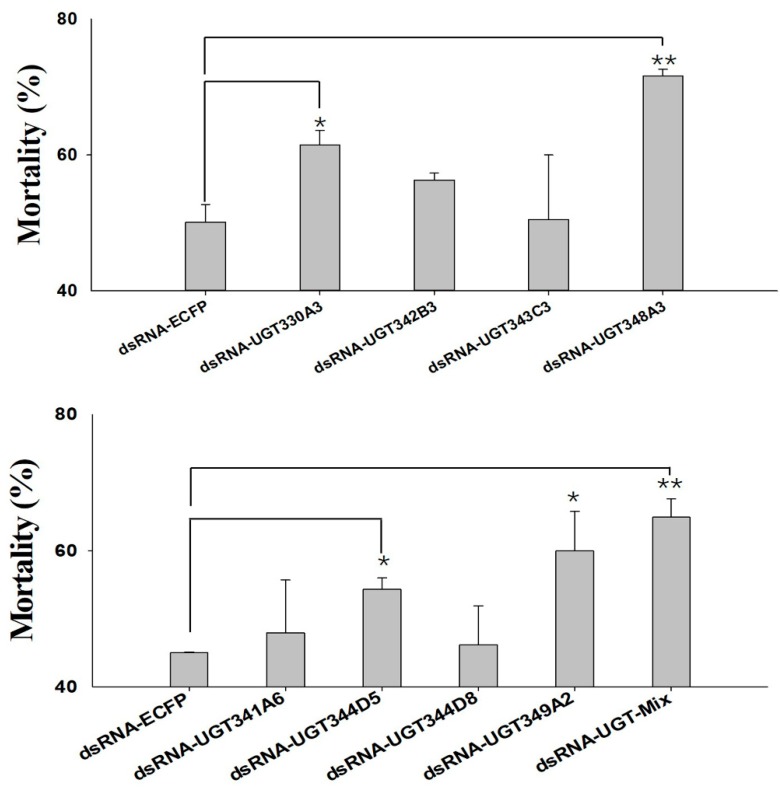
Effects of knockdown of *UGT* transcripts on mortality of *M. persicae nicotianae*. Mean mortality ± SE (*n* = 3) of *M. persicae nicotianae* fed nicotine (100 mg/L) and a dsRNA-*UGT* (100 ng/μL) for 48 h. Each treatment included three replicates, and 80 adult *M. persicae nicotianae* were used in each replicate. * Significant (*p* < 0.05) difference according to Student’s *t*-test. ** Significant (*p* < 0.01) difference according to Student’s *t*-test.

RNAi experiments using orally-delivered dsRNA were performed to elucidate the relationship between downregulation of highly-overexpressed *UGTs* and mortality, which could be a result of nicotine intolerance. Ten *UGT* genes highly expressed in *M. persicae nicotianae* (*UGT330A3*, *UGT339A2*, *UGT341A6*, *UGT342B3*, *UGT343C3*, *UGT344D5*, *UGT344D8*, *UGT348A3*, *UGT349A3*, and *UGT350A3*) according to the qRT-PCRs (Figure 4) were chosen for the RNAi experiments. After dsRNA treatment (100 ng/µL *dsRNA-UGT*), the expression levels of *UGT330A3*, *UGT341A6*, *UGT342B3*, *UGT343C3*, *UGT344D5*, *UGT344D8*, *UGT348A3*, and *UGT349A3* were reduced by 0.50-, 0.54-, 0.46-, 0.58-, 0.34-, 0.40-, 0.64-, and 0.47-fold after 48 h compared to the levels in the control group (Figure 5). Conversely, expression of *UGT339A2* and *UGT350A3* was not affected by oral delivery of dsRNA (Figure 5). Mortality under 100 mg/L nicotine treatment significantly increased after RNAi, from 50.10% in the control group to 61.47% and 71.65% in the *dsRNA-UGT330A3*- and *dsRNA-UGT348A3*-fed groups, respectively (Figure 6). Moreover, suppression of *UGT344D5* and *UGT349A2* expression dramatically increased mortality from 45.03% in the control group to 54.31% and 59.98% in the *dsRNA-UGT344D5*- and *dsRNA-UGT349A2*-treated groups, respectively, and mortality under 100 mg/L nicotine treatment was highest (64.91%) among aphids fed a dsRNA mixture (with equal amounts of the eight *dsRNA-UGT-Mix*) (Figure 6).

## 3. Discussion

Host plants accumulate phytotoxins, such as nicotine in *Nicotiana tabacum*, to resist insect herbivores, and aphid survival in the presence of toxic secondary metabolite nicotine depends on nicotine metabolism. Indeed, metabolism is essential for insects to counter the effects of toxins distributed in host plants. Research has indicated that higher expression of the phase I enzyme P450-*CYP6CY3* in *M. persicae nicotianae* versus *M. persicae* sensu stricto, which is partly due to gene amplification, accounts for the nicotine and neonicotinoid resistance of the subspecies [21]. In the cotton bollworm, larval tolerance to gossypol depends on *CYP6AE14*-mediated detoxification [11,24]. Recent studies have also indicated that the phase II enzymes UGT41B3 and UGT40D1 are capable of glycosylating gossypol to form mainly diglycosylated gossypol isomer 5, accounting for gossypol tolerance in *H. armigera* [20]. In addition, stereoselective reglucosylation of the benzoxazinoid DIMBOA by UGTs represents a detoxification strategy for the adaptation of *Spodoptera* species to benzoxazinoid-containing plants [19]. Although the roles of P450 in nicotine tolerance in *M. persicae nicotianae* have been described [21], the potential roles of UGTs in nicotine tolerance remain unknown. To clarify the roles of these enzymes in host plant adaptation in *M. persicae nicotianae*, two UGT inhibitors, 5-Nul and Sul, were used in a synergism assay. 5-Nul/Sul significantly promoted nicotine toxicity in *M. persicae nicotianae* (Figure 1), illustrating that UGTs may be involved in nicotine tolerance. Therefore, glycosylation by UGTs may play an important role in the detoxification of xenobiotics or the primary metabolites of xenobiotics in *M. persicae nicotianae*. However, information regarding *UGTs* in *M. persicae* is very limited to date.

In this study, 101 *UGT* transcripts were identified from the *M. persicae* genome and transcriptome data. These *UGTs* can be distributed into 11 families, UGT330A3, UGT339A2, UGT341A6, UGT342B3, UGT343C3, UGT344D5, UGT344D8, UGT348A3, UGT349A3, and UGT350A3, with the UGT344 family containing the most transcripts (57 *UGTs*) (Figure 2). The UGT protein structure is divided into two main parts: an aglycone substrate-binding domain in the N-terminus and a UDP sugar donor-binding domain in the C-terminus [23,25]. Alignment of UGT amino acid sequences revealed conserved domains, including sugar donor-binding regions (DBR1 and DBR2), important residues interacting with the sugar donor and catalytic residues, and the UGT signature motif sequence ((FVA)-(LIVMF)-(TS)-(HQ)-(SGAC)-G-X(2)-(STG)-X(2)-(DE)-X(6)-P-(LIVMFA)-(LIVMFA)-X(2)-P-(LMVFIQ)-X(2)-(DE)-Q) (Figure 3) [13]. These results suggest that these UGTs are likely active proteins that function in *M. persicae* as they do in other species.

Insect UGTs are not only capable of detoxifying plant secondary compounds [19,20], some studies have also indicated that UGTs are involved in insecticide resistance [26,27,28]. For example, UGT overexpression is associated with imidacloprid and abamectin resistance in *Leptinotarsa decemlineata* and *Tetranychus cinnabarinus*, respectively [29,30]. However, there has thus far been no large-scale screening of potential xenobiotic tolerance-associated *UGTs* in *M. persicae*. In the present study, qRT-PCR analysis of the transcriptional profiles of *UGTs* based on transcriptome data revealed that the expression levels of ten *UGT* genes was consistent with the levels of nicotine tolerance in the *M. persicae* sensu stricto (green/red morph) and *M. persicae nicotianae* populations (Figure 4), suggesting the potential roles of these *UGTs* in host plant adaptation in *M. persicae nicotianae*. In vivo suppression of *UGT* gene expression by RNAi was employed to examine the functions of *UGTs* in xenobiotic compound resistance. In previous studies, knockdown of highly-expressed *UGT* increased the susceptibility of resistant *Leptinotarsa decemlineata* to imidacloprid [29]. In *Plutella xylostella*, RNAi of transcript *UGT2B17* (renamed *UGT33AA4*) showed this gene to be associated with chlorantraniliprole resistance [26]. To assess the influence of strong *UGT* gene expression on nicotine tolerance in *M. persicae nicotianae*, we performed RNAi via the dsRNA oral feeding method [31]. RNAi of the four highly-expressed *UGT* genes (*UGT330A3*, *UGT344D5*, *UGT348A3*, and *UGT349A3*) alone and a mixture of eight dsRNA of UGTs significantly increased susceptibility to nicotine in *M. persicae nicotianae* (Figure 5 and Figure 6). This result further confirmed that the enzymes encoded by these strongly-expressed *UGTs* (*UGT330A3*, *UGT344D5*, *UGT348A3*, and *UGT349A3*) may contribute to detoxification of nicotine or its primary metabolites via glycosylation and may further contribute to host plant adaptation in *M. persicae nicotianae*. These results further our understanding of host adaptation mechanisms in *M*. *persicae*.

## 4. Materials and Methods

### 4.1. Insects

Three *M. persicae* clone populations were established from a population that was originally collected in 2008 from a field in Jilin Province, China. An *M. persicae* sensu stricto population (green/red morph) was sampled and reared on Chinese cabbage (*Brassica rapa chinensis*). An *M. persicae nicotianae* population (green morph) was collected and reared on tobacco (*N. tabacum* L.). All populations were reared in the laboratory at 20 °C–23 °C with a photoperiod of 16 h:8 h (light:dark).

### 4.2. Synergism Assays

The synergistic effects of the two UGT inhibitors Sul and 5-Nul on the toxicity of nicotine in *M*. *persicae nicotianae* were tested using the artificial diet method, as described by Peng et al. (2016) [31]. The artificial diet (15% sucrose) was sealed between two layers of Parafilm in a 4-cm feeding arena. Aphids were placed in the arena, which was covered with fine mesh to prevent their escape. The maximum sublethal doses of Sul/5-Nul in *M*. *persicae nicotianae* were determined using the oral feeding method. At least five different concentrations of Sul/5-Nul and a control were used. The maximum dose that led to zero mortality in *M*. *persicae nicotianae* was adopted as the maximum sublethal concentration in our study. Eighty apterous adult *M. persicae nicotianae* were fed an artificial diet that contained nicotine (final concentration: 100 mg/L) with or without added Sul/5-Nul (final concentration: 12.5 mg/L); the artificial diet without inhibitor was used as a control. Each treatment included three replicates (80 apterous adults in each replicate). Mortality was recorded after 48 h. Synergistic effects were calculated by comparing the corrected mortality without Sul/5-Nul to the corrected mortality with Sul/5-Nul.

### 4.3. Nomenclature and Phylogenetic Analysis

*UGT* sequences from the genome of *M. persicae* in NCBI and the transcriptome of *M. persicae* (the SRA experiment Accession Number SRX1499035) were obtained [31]. These *UGT* sequences were named according to the UGT Nomenclature Committee (http://prime.vetmed.wsu.edu/resources/udp-glucuronsyltransferase-homepage) guidelines to include the following components: the symbol *UGT*, a family number, a subfamily letter, and an individual gene number. *UGT* families are defined as having 40% amino acid sequence identity, and subfamilies are defined as having 60% or greater amino acid identity [23]. Names were assigned to the *M. persicae UGT* sequences on this basis (Supplementary Data 1). Predicted *UGT* protein sequences from *H. armigera* and *B. mori* (Supplementary Data 2) were extracted from UGT Nomenclature Committee resources and aligned against *M. persicae* UGTs using ClustalW in MEGA7 software (http://www.megasoftware.net/). These alignments were used to build a consensus phylogenetic tree with the neighbor-joining method. Pairwise and multiple alignments were performed with a gap opening penalty of 10 and a gap extension penalty of 0.2. A total of 1000 bootstrap replicates were used. Branches with bootstrap values above 50% are indicated.

### 4.4. Protein Structure Prediction

Multiple alignments of nine highly-expressed representative protein sequences among the *M*. *persicae nicotianae* UGTs were performed using ClustalW, and structural domains such as the signature UGT motif were detected by comparison with other sequences with characterized primary structures. Signal peptides were predicted by SignalP 4.1 on CBS Prediction Servers (http://www.cbs.dtu.dk/services/SignalP/). The C-terminal transmembrane domain was identified with TMHMM2.0 (http://www.cbs.dtu.dk/services/TMHMM).

### 4.5. Total RNA Isolation and cDNA Synthesis

Total RNA was extracted from apterous adult aphids with TRIzol (Invitrogen, Carlsbad, CA, USA) according to the manufacturer’s instructions and treated with RNase-free DNase I (TaKaRa, Kyoto, Japan). The RNA samples were quantified by measuring absorbance at 260 nm; quality was assessed via agarose gel electrophoresis. First-strand cDNA was synthesized from total RNA using a PrimeScript^TM^ First-Strand cDNA Synthesis Kit (Takara, Japan) with oligo(dT)_18_.

### 4.6. qRT-PCR and Data Analysis

qRT-PCR was performed using an ABI 7500 instrument (Applied Biosystems) with SYBR^®^ Premix Ex Taq™ II (Tli RNaseH Plus; Takara, Japan). The gene primers for qRT-PCR (Table S1) were synthesized by Sangon Biotech Co., Ltd. (Shanghai, China). The thermal cycling protocol included an initial denaturation step at 95 °C for 30 s followed by 40 cycles of 95 °C for 5 s and 60 °C for 34 s. The fluorescence signal was measured at the end of each extension step at 60 °C. After amplification, a dissociation program with steps of 95 °C for 15 s, 60 °C for 1 min, and 95 °C for 15 s was performed to confirm that only specific products were amplified. The housekeeping genes actin and *para*, which encodes a voltage-gated sodium channel, were used as internal reference genes for *M. persicae nicotianae* [32]. Relative gene expression was calculated with the 2^−ΔΔCT^ method [33]. The experiment was independently performed three times for each strain. Significant differences were analyzed using GraphPad InStat 3 statistical software (GraphPad Software, 2000).

### 4.7. Artificial Diet Rearing and dsRNA Feeding

Using DNAMAN 6.0 software, we designed specific primers based on the *UGT* sequences of *M. persicae* (Supplementary Data 1) and the possible interference sites predicted with online prediction software (http://www.dkfz.de/signaling/e-rnai3/). Gene fragments were amplified from cDNA and cloned into the pGEM-T vector (Promega, Madison, WI, USA). The amplified DNA fragments served as templates for RNA synthesis using the T7 RiboMAX™ Express RNAi System (Promega, USA). Enhanced cyan fluorescent protein (*ECFP*) dsRNA was synthesized under identical conditions with the primers listed in Table S1. The artificial diet and the rearing method used in this study were reported previously by Peng et al. (2016) [31]. The diet (15% sucrose) was prepared in DEPC-treated water to ensure the absence of RNase activity. For the dsRNA feeding experiments, dsRNA was added to the artificial diet at a concentration of 100 ng/μL; an artificial diet containing dsRNA-*ECFP* was used as a control. Eighty apterous adult *M. persicae nicotianae* were transferred onto the artificial diet for rearing. For analysis of the efficiency of dsRNA knockdown of *UGT* expression, the aphids were fed an artificial diet containing dsRNA (100 ng/μL) for 48 h and then collected for qRT-PCR. To assess the sensitivity of the aphids to nicotine after RNAi of *UGT*, 80 apterous adult *M. persicae nicotianae* individuals were transferred to an artificial diet (1 mL) containing nicotine (100 mg/L) mixed with dsRNA-*UGT* (100 ng/μL); dsRNA-*ECFP* was used as the control. The mortality of the aphids was recorded after 48 h. Each treatment included three replicates (80 aphids in each replicate).

## 5. Conclusions

The present study confirmed that the enzymes encoded by the identified highly-expressed *UGTs* may contribute to detoxification of nicotine or its primary metabolites by glycosylation and may further contribute to host plant adaptation in *M. persicae nicotianae*. These results contribute to our understanding of host adaptation mechanisms in *M*. *persicae*.

## Supplementary Materials

The following are available online at https://www.mdpi.com/1422-0067/20/15/3637/s1. Table S1: Primers used in the experiments. Act: actin; Aph: *para* (a voltage-gated sodium channel); dsRNA, double-stranded RNA; F, forward; ORF, open reading frame; R, reverse. Lowercase letters indicate the T7 RNA polymerase promoter. Supplementary Data 1: *M. persicae UGT* transcripts. Supplementary Data 2: *H. armigera* and *B. mori UGT* sequences extracted from the UGT Nomenclature Committee database.

## Abbreviations

UGT	Uridine diphosphate (UDP)-glycosyltransferase
P450	Cytochrome P450 monooxygenase
CarE	Carboxylesterase
GST	Glutathione S-transferase
qPCR	Quantitative real-time polymerase chain reaction
RNAi	RNA interference
Sul	Sulfinpyrazone
5-Nul	5-nitrouracil
NCBI	National Center for Biotechnology Information
SRA	Sequence Read Archive database
ECFP	Enhanced cyan fluorescent protein
DEPC	Diethyl pyrocarbonate
dsRNA	Double-stranded RNA
3D	Three-dimensional
DBR	Sugar donor-binding regions
PSPG	Plant secondary product glycosyltransferase motif
