# Supplementary material for: Characterization of UDP-Glucuronosyltransferases and the Potential Contribution to Nicotine Tolerance in Myzus persicae
Source: Int J Mol Sci. 2019 Jul 25;20(15):3637. doi: 10.3390/ijms20153637 (PMC6695686; doi:10.3390/ijms20153637)
Supplement: Supplementary file 1 [file ijms-20-03637-s001.zip › Table S1.pdf]

Table S1. Primers used in experiments.

Primer name	Sequence (5'-3')	Application	Fragment length(bp)
ActF	GGTGTCTCACACACAGTGCC	Real-Time PCR	131
ActR	CGGCGGTGGTGGTGAAGCTG	Real-Time PCR	
AphF	TGGTATACACGTTGGTTCTC	Real-Time PCR	132
AphR	GACCACGAGCTTCCCCGGTG	Real-Time PCR	
UGT348A3-F	GGTCGTATCCATTCCCATC	Real-Time PCR	173
UGT348A3-R	CAGTGGCGAAACTCCTACA	Real-Time PCR	
UGT344C6-F	CATCAGTAAACTAAAACCTAAA	Real-Time PCR	98
UGT344C6-R	CAGCCACAGTGTCCCATC	Real-Time PCR	
UGT344N3-F	TTTTCATTTATTGCTCCTG	Real-Time PCR	88
UGT344N3-R	GGAATCGGTTATAGTAAGGT	Real-Time PCR	
UGT344H2-F	GTAGCGAAGTGGCTGTTGA	Real-Time PCR	131
UGT344H2-R	CTGTTGGCGTACAGTGTGTT	Real-Time PCR	
UGT344B8-F	CATAAAGGCATTGGCTGG	Real-Time PCR	140
UGT344B8-R	AAATAGTGTTGGGGGGAG	Real-Time PCR	
UGT350A3-F	TATTCATTCAGATGGCTAT	Real-Time PCR	90
UGT350A3-R	GCACTAAACTTATGGCTC	Real-Time PCR	
UGT343A6-F	AGTTTTGTGTATCGCAGCC	Real-Time PCR	227
UGT343A6-R	TGACATTTCTCAGCCTTCC	Real-Time PCR	
UGT344L3-F	CGGGAAATGGTGCGAGAA	Real-Time PCR	134
UGT344L3-R	ACCGTCAAGGAATGGCGT	Real-Time PCR	
UGT329B4-F	AGACATTGCCTCCGAACA	Real-Time PCR	153
UGT329B4-R	TATTTTTCTCCGTTGCTCA	Real-Time PCR	
UGT350C4-F	ATGGATTACCGTTTACAG	Real-Time PCR	104
UGT350C4-R	GGTAGAAACACCAGAATG	Real-Time PCR	
UGT343C3-F	TTCCTTACACTATTCACTGC	Real-Time PCR	171
UGT343C3-R	GTAGTTAGGCTGTGGTGTC	Real-Time PCR	
UGT339A2-F	TATGGTGTTGTTGCTGGA	Real-Time PCR	83
UGT339A2-R	AGTGACTGTGACCGTTGTA	Real-Time PCR	
UGT330A3-F	GTTTCGGAAGCCCTGACG	Real-Time PCR	72
UGT330A3-R	TTCCTTCGGGGACTTGGG	Real-Time PCR	
UGT343A5-F	ACCGTGGTCAGTTCATTC	Real-Time PCR	85
UGT343A5-R	TTCTCCCTCTTGTTTTGTC	Real-Time PCR	
UGT342B3-F	GTCACACACTTGTTGGGC	Real-Time PCR	82
UGT342B3-R	CAGATGTAGTAACGGCGA	Real-Time PCR	
UGT349A3-F	GTTCCCTCAGAAAACGCCTC	Real-Time PCR	212
UGT349A3-R	TGATGCCGCTTTGGAGGA	Real-Time PCR	
UGT343B3-F	AGGAGTTGGCTCGTCGTG	Real-Time PCR	194

UGT343B3-R	GGACAAGAGACGGGCGAA	Real-Time PCR	
UGT350C5-F	ATCAGCAGGGTAACTCACTC	Real-Time PCR	118
UGT350C5-R	GTCGGATCAATGAGGAGC	Real-Time PCR	
UGT329A5-F	TTTTTTTATTTGTAGCAGTA	Real-Time PCR	97
UGT329A5-R	TGACTAAAGCCTTGATGAG	Real-Time PCR	
UGT329A4-F	TGTTCTTGCCTTACCTGCG	Real-Time PCR	93
UGT329A4-R	TGTCGGTAATGTTCGGGTG	Real-Time PCR	
UGT344M3-F	AACAGTTCGGGTCGGTGGT	Real-Time PCR	157
UGT344M3-R	TAAAACGGCTCAAGCAGGA	Real-Time PCR	
UGT344M4-F	CATCCCCACAGTATCTCCG	Real-Time PCR	163
UGT344M4-R	TTTCCGTGTAGTTCTCCCG	Real-Time PCR	
UGT342C3-F	AGCATCACCTTGGGAAACG	Real-Time PCR	171
UGT342C3-R	GAGGCAAGCAACTCCGTGA	Real-Time PCR	
UGT344F5-F	GTTTACACCCCGTTCCCG	Real-Time PCR	100
UGT344F5-R	TAGTGGCGTCCGTGTGGT	Real-Time PCR	
UGT344F4-F	CGAAGTGCCCCTCGTGTA	Real-Time PCR	141
UGT344F4-R	AGTCGCCTGGCTAAAGTGTC	Real-Time PCR	
UGT344E7-F	GTTTACACCATTTCCTGA	Real-Time PCR	157
UGT344E7-R	TAAGTTACGATTAGTCCTC	Real-Time PCR	
UGT345A3-F	TTGTCAACACGCATCACTC	Real-Time PCR	125
UGT345A3-R	GTCTATCACATCTGCTAAATCTC	Real-Time PCR	
UGT341A5-F	TGACAATCTTTGGTAGCGG	Real-Time PCR	159
UGT341A5-R	AAAAAGGGCTGACTACGGT	Real-Time PCR	
UGT341A6-F	CCACCACTCCGTATCGTA	Real-Time PCR	146
UGT341A6-R	TGAAGTAGATCGCCCCAT	Real-Time PCR	
UGT344B6-F	TATGTTTGCTTGTTCGTG	Real-Time PCR	85
UGT344B6-R	CCATTGACTTTTACCTCCT	Real-Time PCR	
UGT344A12-F	AATGGCACCGATTGTTAGC	Real-Time PCR	105
UGT344A12-R	CACAGCCCGACCGAGTAG	Real-Time PCR	
UGT344B7-F	CTCGTGGATACCTGCCCC	Real-Time PCR	108
UGT344B7-R	TCCACCAATGCCCGAAGA	Real-Time PCR	
UGT344A13-F	GCCGTTCATAGATGGGGAC	Real-Time PCR	158
UGT344A13-R	CTCCACAGTTGGTCTTAGTCG	Real-Time PCR	
UGT344D5-F	ATGGCGACGACGAAGAGA	Real-Time PCR	187
UGT344D5-R	GCGTGAAAACCGTGACCT	Real-Time PCR	
UGT344D7-F	ACTTGATGGCTTCGGGGG	Real-Time PCR	358
UGT344D7-R	TTCTGGAAGATGATGGTGGG	Real-Time PCR	
UGT344D8-F	GGTATACGGCAACCAAAAA	Real-Time PCR	94
UGT344D8-R	ACAGTCAAAGCCCAACGG	Real-Time PCR	
UGT344D9-F	CGACAATGCTCACCTTCTCT	Real-Time PCR	120
UGT344D9-R	TGTTGGCGAACCTCTGGAC	Real-Time PCR	

UGT348A3-dsF1	GGTGtaatacgactcactataggAGTATTAGTTTATGGGAAGGGG	dsRNA synthesis	554
UGT348A3-dsR1	CGGACTGTAGGAGTTTCGC	dsRNA synthesis	
UGT348A3-dsF2	AGTATTAGTTTATGGGAAGGGG	dsRNA synthesis	
UGT348A3-dsR2	GGTGtaatacgactcactataggCGGACTGTAGGAGTTTCGC	dsRNA synthesis	
UGT330A3-dsF1	GGTGtaatacgactcactataggAACGGTTACCTGAAAGAGATGC	dsRNA synthesis	712
UGT330A3-dsR1	CGTCCTCCCATTTCCACAA	dsRNA synthesis	
UGT330A3-dsF2	AACGGTTACCTGAAAGAGATGC	dsRNA synthesis	
UGT330A3-dsR2	GGTGtaatacgactcactataggCGTCCTCCCATTTCCACAA	dsRNA synthesis	
UGT339A2-dsF1	GGTGtaatacgactcactataggCACGCTTCCGCCAGTCATCA	dsRNA synthesis	681
UGT339A2-dsR1	CCCTTCAACAACGAACCAAA	dsRNA synthesis	
UGT339A2-dsF2	CACGCTTCCGCCAGTCATCA	dsRNA synthesis	
UGT339A2-dsR2	GGTGtaatacgactcactataggCCCTTCAACAACGAACCAAA	dsRNA synthesis	
UGT350A3-dsF1	GGTGtaatacgactcactataggATTCTGGGTACAAAGATCACA	dsRNA synthesis	525
UGT350A3-dsR1	TAGGGTGCCCAAGAATGTC	dsRNA synthesis	
UGT350A3-dsF2	ATTCTGGGTACAAAGATCACA	dsRNA synthesis	
UGT350A3-dsR2	GGTGtaatacgactcactataggTAGGGTGCCCAAGAATGTC	dsRNA synthesis	
UGT349A3-dsF1	GGTGtaatacgactcactataggAAATCACCCAAGCCCATAC	dsRNA synthesis	587
UGT349A3-dsR1	GTTCTCAAGTGTTTCGCCC	dsRNA synthesis	
UGT349A3-dsF2	AAATCACCCAAGCCCATAC	dsRNA synthesis	
UGT349A3-dsR2	GGTGtaatacgactcactataggGTTCTCAAGTGTTTCGCCC	dsRNA synthesis	
UGT343C3-dsF1	GGTGtaatacgactcactataggAATCTTACCGTAGTCAGCAC	dsRNA synthesis	617
UGT343C3-dsR1	GGTCTTGTTACACCGATACTG	dsRNA synthesis	
UGT343C3-dsF2	AATCTTACCGTAGTCAGCAC	dsRNA synthesis	
UGT343C3-dsR2	GGTGtaatacgactcactataggGGTCTTGTTACACCGATACTG	dsRNA synthesis	
UGT342B3-dsF1	GGTG taatacgactcactataggATCGCCGTTACTACATCTG	dsRNA synthesis	712
UGT342B3-dsR1	CGTTTGCTATGTTTCTGTGT	dsRNA synthesis	
UGT342B3-dsF2	ATCGCCGTTACTACATCTG	dsRNA synthesis	
UGT342B3-dsR2	GGTGtaatacgactcactataggCGTTTGCTATGTTTCTGTGT	dsRNA synthesis	
UGT344D5-dsF1	GGTGtaatacgactcactataggCGCAACATTCGTCCAGAGG	dsRNA synthesis	454
UGT344D5-dsR1	TTCTCGTTGAGGAAACCATT	dsRNA synthesis	
UGT344D5-dsF2	CGCAACATTCGTCCAGAGG	dsRNA synthesis	
UGT344D5-dsR2	GGTGtaatacgactcactataggTTCTCGTTGAGGAAACCATT	dsRNA synthesis	
UGT344D8-dsF1	GGTGtaatacgactcactataggCGTTGGGCTTTGACTGTGT	dsRNA synthesis	575
UGT344D8-dsR1	CGGTTTGTCCTTCATTTCG	dsRNA synthesis	
UGT344D8-dsF2	CGTTGGGCTTTGACTGTGT	dsRNA synthesis	
UGT344D8-dsR2	GGTGtaatacgactcactataggCGGTTTGTCCTTCATTTCG	dsRNA synthesis	
UGT341A6-dsF1	GGTGtaatacgactcactataggTAAAGATGTCGGACCTGGG	dsRNA synthesis	502
UGT341A6-dsR1	GGCAATTTGTAATCTGCAACA	dsRNA synthesis	
UGT341A6-dsF2	TAAAGATGTCGGACCTGGG	dsRNA synthesis	
UGT341A6-dsR2	GGTGtaatacgactcactataggGGCAATTTGTAATCTGCAACA	dsRNA synthesis	
DsECFP-F1	GGTGtaatacgactcactataggTTACGCCAAGCTTGCATGCCT	dsRNA synthesis	732

DsECFP-R1	ACTCCAGCAGGACCATGTGATC	dsRNA synthesis
DsECFP-F2	TTACGCCAAG CTTGCATGCCT	dsRNA synthesis
DsECFP-R2	GGTGtaatacgactcactataggACTCCAGCAGGACCATGTGATC	dsRNA synthesis

Act: actin; Aph: *para* (a voltage-gated sodium channel); dsRNA, double-stranded RNA; F, forward; ORF, open reading frame; R, reverse. Lower-case indicates the T7 RNA polymerase promoter.
